# Modeling the His-Purkinje Effect in Non-invasive Estimation of Endocardial and Epicardial Ventricular Activation

**DOI:** 10.1007/s10439-022-02905-4

**Published:** 2022-01-24

**Authors:** Machteld J. Boonstra, Rob W. Roudijk, Rolf Brummel, Wil Kassenberg, Lennart J. Blom, Thom F. Oostendorp, Anneline S. J. M. te Riele, Jeroen F. van der Heijden, Folkert W. Asselbergs, Peter Loh, Peter M. van Dam

**Affiliations:** 1 Division Heart & Lungs, Department of Cardiology, University Medical Center Utrecht, Utrecht University, 3508 GA Utrecht, The Netherlands; 2 Netherlands Heart Institute, Utrecht, The Netherlands; 3 Donders Institute for Brain, Cognition and Behaviour, Radboud University Nijmegen Medical Centre, Nijmegen, The Netherlands; 4 Institute of Cardiovascular Science, Faculty of Population Health Sciences, University College London, London, UK; 5 Health Data Research UK and Institute of Health Informatics, University College London, London, UK

**Keywords:** Cardiovascular imaging, Electro-anatomical mapping, Inverse electrocardiography, Electrocardiography, Electrocardiographic imaging, Electrophysiology, Equivalent dipole layer, Non-invasive cardiac activation mapping, His-Purkinje system

## Abstract

Inverse electrocardiography (*i*ECG) estimates epi- and endocardial electrical activity from body surface potentials maps (BSPM). In individuals at risk for cardiomyopathy, non-invasive estimation of normal ventricular activation may provide valuable information to aid risk stratification to prevent sudden cardiac death. However, multiple simultaneous activation wavefronts initiated by the His-Purkinje system, severely complicate *i*ECG. To improve the estimation of normal ventricular activation, the *i*ECG method should accurately mimic the effect of the His-Purkinje system, which is not taken into account in the previously published multi-focal *i*ECG. Therefore, we introduce the novel multi-wave *i*ECG method and report on its performance. Multi-wave *i*ECG and multi-focal *i*ECG were tested in four patients undergoing invasive electro-anatomical mapping during normal ventricular activation. In each subject, 67-electrode BSPM were recorded and used as input for both *i*ECG methods. The *i*ECG and invasive local activation timing (LAT) maps were compared. Median epicardial inter-map correlation coefficient (CC) between invasive LAT maps and estimated multi-wave *i*ECG versus multi-focal *i*ECG was 0.61 versus 0.31. Endocardial inter-map CC was 0.54 respectively 0.22. Modeling the His-Purkinje system resulted in a physiologically realistic and robust non-invasive estimation of normal ventricular activation, which might enable the early detection of cardiac disease during normal sinus rhythm.

**Supplementary Information:**

The online version contains supplementary material available at 10.1007/s10439-022-02905-4.

## Introduction

Recorded body surface potential maps (BSPM) have a direct relation to the cardiac electrical activity. Methods to describe this relation are often referred to as ‘solving the inverse problem of electrocardiography’, ‘epicardial electrocardiographic (ECG) imaging’ or ‘inverse electrocardiography (*i*ECG)’. The most commonly used method is based on the Equivalent Potential Distribution (EPD) model.^2,29^ In this method, the cardiac activation pattern is estimated by solving the mathematical linear relation between the electrical activity on the body surface and the epicardial surface. Recently this method has also been adjusted to estimate both endocardial and epicardial activation. Later, Equivalent Double Layer (EDL)-based *i*ECG was introduced, relating body surface electrical activity to electrical activity both on the endocardium and the epicardium by simulating the generated cardiac currents.^11,35^ The currents generated by the heart follow the local transmembrane potential waveform, thereby creating a direct link to cardiac electrophysiology. Furthermore, EDL-based *i*ECG requires an initial estimate that can be based on ventricular electrophysiology, in contrast to EPD-based *i*ECG.^17,35^

**Figure 1:**
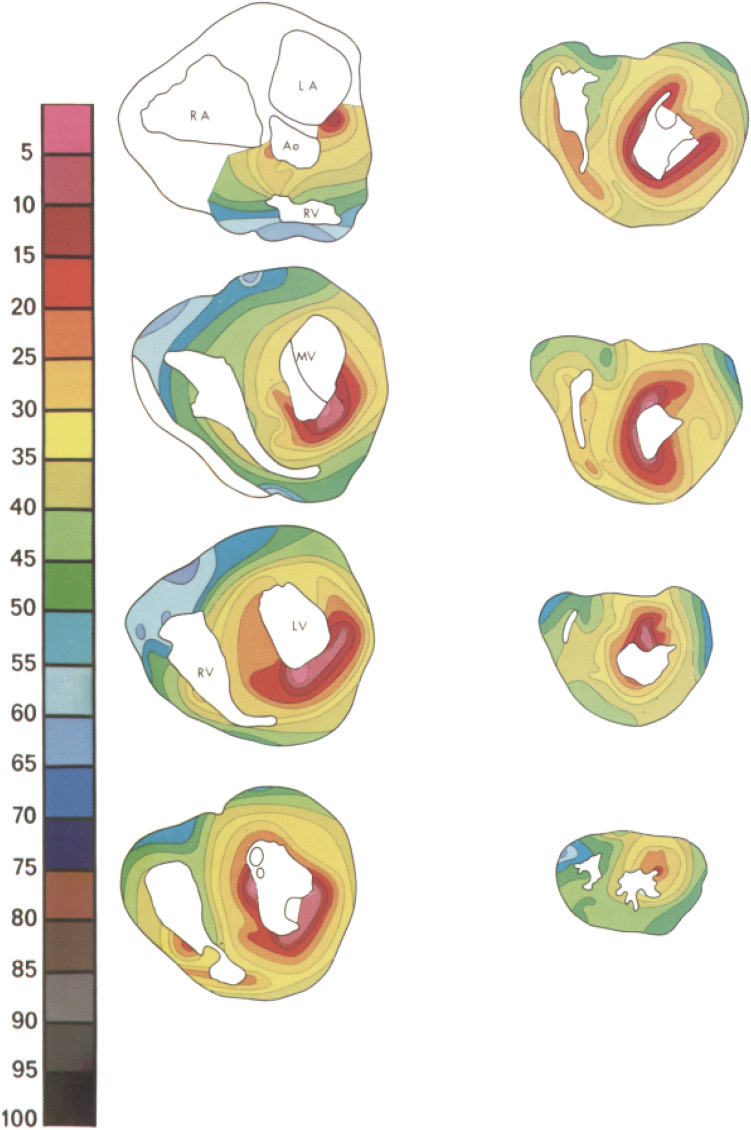
Normal ventricular activation in a healthy subject. In eight slices ventricular activation is displayed from early (pink) to late (blue). Around the endocardial structures in the LV (papillary muscles), around the RV moderator band, at the LV septal wall and near the MV, areas of early activation are observed. *LA* left atrium, *RA* right atrium, *Ao* aortic valve, *RV* right ventricle, *MV* mitral valve, *LV* left ventricle. Reproduced with permission from Refs. 10 and 34

Inverse estimation of cardiac activity has been used to determine origins of arrhythmias^23,32^ or to provide insight in electrophysiological substrates of structurally diseased hearts.^1,28^ In individuals with genetic predisposition for cardiomyopathies, ventricular arrhythmias or sudden cardiac death can be the first manifestation of disease. In these individuals, adequate non-invasive identification of the arrhythmogenic substrate during normal His-Purkinje initiated ventricular activation, may prove to be of utmost importance to improve early detection of disease and aid early treatment. However, the non-invasive estimation of normal ventricular activation using BSPM is challenging, due to the nature of this complex activation pattern. Ventricular activation initiated by the His-Purkinje system is the result of multiple near simultaneous activation waves initiated at several endocardial locations (Fig. 1).^10,20,40^ Recorded BSPM waveforms are the result of spatial summation of the current generated by these simultaneous activation wavefronts, resulting in partial cancellation and amplification.^10^ Therefore, the non-invasive estimation of normal ventricular activation is more complicated than of ventricular activation from a single focus such as premature ventricular contractions. Studies of the anatomy of the His-Purkinje system^16,18,34^ and the Purkinje-myocardial junctions^25,33^ showed inter-individual diversity and structural heart disease or conduction defects complicate the non-invasive estimation of normal ventricular activation even more.^6,15^ Furthermore, the His-Purkinje system consists of numerous ramifications and terminates in Purkinje myocardial junctions distributed over a large part of the ventricular endocardium. However, the endocardium is not activated simultaneously as the dense distribution of Purkinje-myocardial junctions may suggest, but by multiple wavefronts initiated at distinct endocardial regions (Fig. 1).^16,18,34^ Characterization of the patient specific anatomy of the His-Purkinje system and the effect of the increased velocity of the sub-endocardial layer has been studied previously in computer models, indicating the effect of these parameters on QRS derived parameters.^5,12,24,39^ In our study, we incorporated a generic model of the His-Purkinje system in the *i*ECG method, as, in line with the findings of the previous studies, this would provide a physiologically realistic estimation of the cardiac activation sequence.

**Figure 2:**
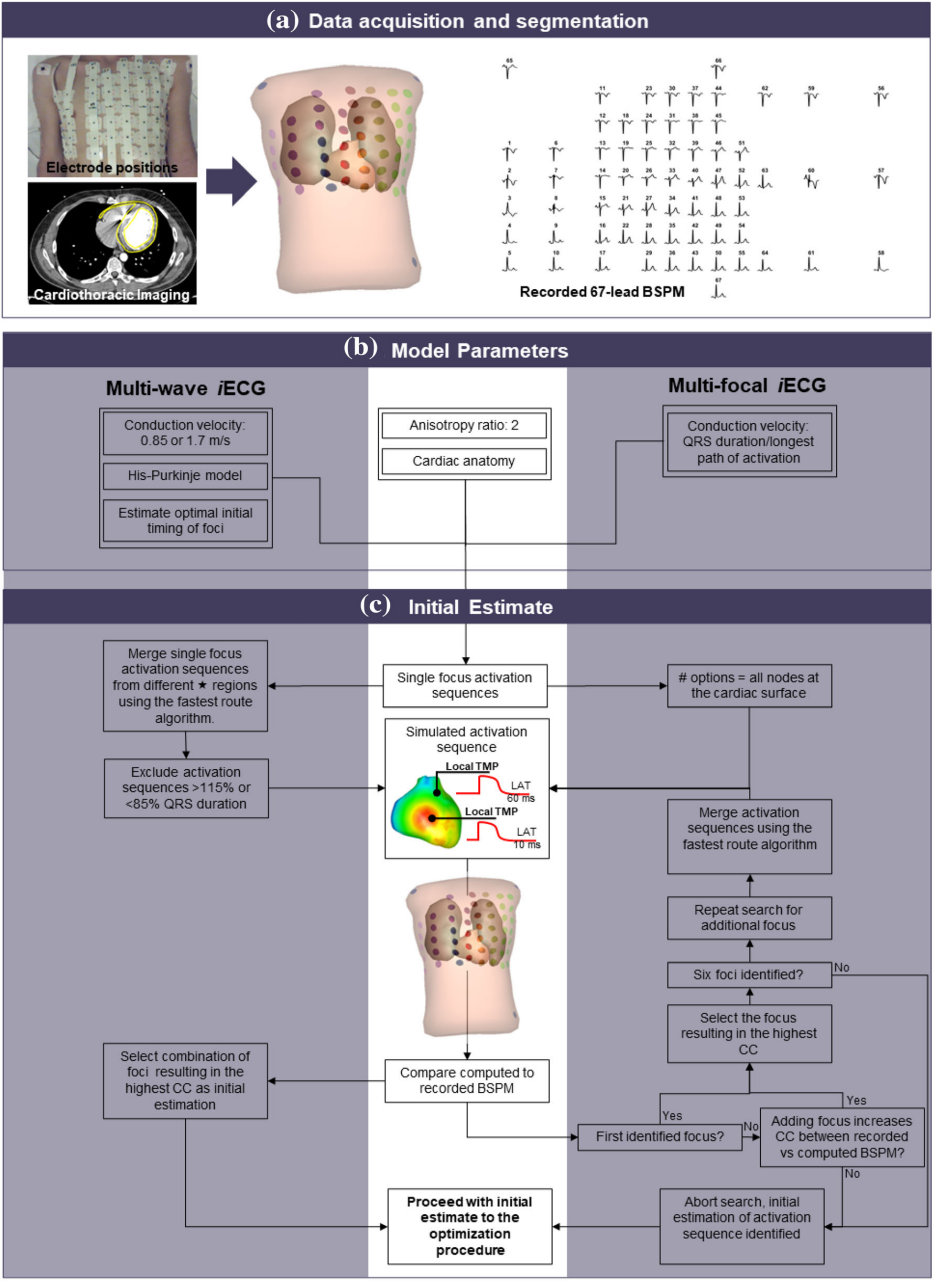
Panel A: Multi-wave *i*ECG. A: (cardiac) imaging and body surface potential maps (BSPM) data are obtained. B: The cardiac source model uses local transmembrane potentials (TMP) to determine activation timing, and using the volume conductor, BSPM are computed. C: Multi-wave *i*ECG tests 511 combinations of the activation sequences and selects the activation sequence with the best matching BSPM based on correlation and QRS duration and D: local activation timing (LAT) maps are computed (one example shown). LAT maps are displayed from red (early) to blue (late). Panel B: Invasive vs *i*ECG comparison: A: The invasive anatomical anatomy was merged with CT based anatomy of the iECG procedure. Invasively obtained LAT were projected onto the iECG anatomy. B: Quantitative comparison between the invasive LAT maps and iECG maps was performed per invasively mapped surface.

In earlier EDL-based studies, the existence of multiple breakthroughs was mimicked through an iterative multi-foci search over the complete ventricular myocardium. However, this method was primarily designed to model focal ventricular activation.^17,23,35^ In this study, we introduce a model for the His-Purkinje system, the multi-wave *i*ECG method, to improve the non-invasive estimation of normal ventricular activation. To this end, we incorporated physiological and anatomical information about the His-Purkinje system in our model wherein the effect of the His-Purkinje system on ventricular activation is mimicked by incorporating anatomical structures associated with early activation (Fig. 2). We then evaluate the performance of multi-wave *i*ECG is and compare it to the previously described multi-focal *i*ECG using patient specific invasive electro-anatomical mapping (EAM).

## Materials and Methods

### Study Population

Four subjects referred for a clinical endocardial and epicardial electro-anatomical mapping (EAM) procedure were studied. Informed consent was obtained for each subject and all underwent *i*ECG as described below.

### Data Acquisition

Each subject underwent imaging, BSPM and invasive endocardial and epicardial EAM. Clinical cardiac imaging was performed whereof subject specific geometries of the complete ventricular myocardium, the left ventricular blood pool, the right ventricular blood pool, the thorax and the lungs were created (Figs. 2A, 2B). The surface of the models of the segmented geometries was discretized by a closed triangulated surface meshes and created by dedicated software (GeomPEACS).^37^ The effect of the volume conductor model was computed using the boundary element method, previously described.^11,21^ Assigned conductivity values were 0.2 S/m for the thorax and ventricular muscles, 0.04 S/m for the lungs and 0.6 S/m for the blood cavities. Electrode positions on the thorax were reconstructed by aligning thorax geometries to 3D images of the thorax with electrodes.^27^ Electro-anatomical structures associated with His-Purkinje mediated activation were incorporated in the imaging-based ventricular geometries (Fig. 2B) as differences in location are known to affect QRS morphology.^14,19,30^

Prior to the invasive procedure, 67-electrode BSPM were recorded (sampling frequency 2048 Hz, Fig. 2A). Recorded BSPM signals were resampled to 1000 Hz and baseline drift and 50Hz noise were removed. Per subject, five subsequent normal ventricular beats were selected and used as input for both *i*ECG methods. Premature ventricular contractions were excluded from analysis.

Invasive EAM was performed under general anesthesia during sinus rhythm using commercial EAM systems (Carto or Ensite Precision) using multi-electrode catheters (PENTARAY® or HD Grid). Right ventricular endocardial access was obtained through the right femoral vein and left ventricular access was gained through transseptal puncture using a steerable sheath. Epicardial access was obtained by percutaneous subxiphoid puncture, thereby puncturing the pericardium. Local unipolar and bipolar contact electrograms at the endocardial and the epicardial ventricular surface were sequentially recorded during normal ventricular activation with simultaneous measurement of the 12-lead ECG. The 12-lead ECG recorded during EAM was used as time-reference for both the BSPM signals and recorded electrograms. After the procedure, measured electrograms and LAT were manually checked for validity. The LAT was set at the maximal absolute amplitude of the bipolar signal, corresponding to the maximum downslope (dV/dt) in unipolar signals, and taking into account neighboring measurements. Data were exported as raw electrograms with annotated LAT, local bipolar voltage and location.

#### Alignment of Ventricular Anatomical Models from CT and EAM

Subject specific imaging-based ventricular *i*ECG geometries were aligned to subject specific EAM point clouds in MATLAB using endocardial anatomical landmarks. The alignment was optimized using a rigid iterative least squares closest point matching algorithm.^3^ LAT and local bipolar voltages were projected onto the nearest triangular surface of the imaging-based ventricular geometry. EAM points >10 mm from the imaging-based ventricular geometry were excluded from analysis. After projection, LAT and local bipolar voltage were averaged on each node of the imaging-based ventricular geometries.

### iECG Method

EDL-based *i*ECG consists of two steps as the relation between activation time and simulated transmembrane potentials is non-linear. First, the required initial estimation of the ventricular activation sequence is computed and in the second step, local activation timings are mathematically optimized by minimizing the differences between recorded and computed BSPM by tuning LAT. In this study, two methods to determine the initial estimation were compared; multi-wave *i*ECG and multi-focal *i*ECG (Fig. 2). In the initial estimation step, different activation sequences are simulated and corresponding BSPM are computed and directly compared to recorded BSPM. The ventricular anatomical model served as the source model (EDL) and at each node, the local transmembrane potentials were simulated which determined the local source strength. Resulting BSPM were computed per simulated activation sequence.^38^

In short, the multi-wave *i*ECG procedure provided an estimation by mimicking the effect of the His-Purkinje system on ventricular activation, multi-focal *i*ECG did not. In both methods, ventricular activation sequences with multiple distinct foci and initial activation timings were estimated using the fastest route algorithm (FRA).^35,36^ For the simulation of activation sequences in both methods, an anisotropy ratio of two was used, meaning that the conduction velocity perpendicular to the myocardial fibers was two times lower than conduction velocity longitudinal to myocardial fibers.^7,13,31^ BSPM were then computed using the boundary element method to determine to compare to the recorded BSPM.^11,21^ The activation sequence yielding highest correlation between recorded and computed BSPM was assumed to be the activation sequence best explaining the recorded BSPM and was selected as the initial estimation and used as input for the optimization procedure. Both methods to estimate the initial activation sequence are described in more detail below.

#### Multi-focal iECG: Adaptation of Principal Single Focus Activation

Multi-focal *i*ECG has been described in several studies.^17,23,35^ In short, this method serves an additive approach based on the FRA. First the ‘fundamental activation sequence’ originating from one focus that achieves the highest correlation between recorded BSPM and computed BSPM is determined. Therefore, myocardial conduction velocity was set by matching the total activation duration of the ‘fundamental’ activation sequence to the QRS duration up to a maximum of 2.5 mm/ms. After determining this fundamental activation sequence, up to six foci are iteratively added if adding improves the match between recorded and computed BSPM (Fig. 2, multi-focal iECG).

#### Multi-wave iECG: Modeling the Effect of the His-Purkinje System

In the novel multi-wave *i*ECG method, the effect of His-Purkinje mediated ventricular activation is mimicked by combining activation sequences initiated at endocardial regions associated with the His-Purkinje system. Several endocardial regions are associated with early ventricular activation; the bases of the two left ventricular papillary muscles, the right ventricular moderator band and several septal regions (Figs. 1 and 2).^6,10,15,16,18,25,33,34,40^ Of note are the two breakthroughs at the LV septal wall, near the mitral valve and near the LV apex. All regions correspond to Purkinje anatomy and observed regions of early activation in more recent invasive mapping studies.^6,10,15,16,18,25,33,34,40^

These anatomical regions were localized in the subject specific imaging-based ventricular geometries based on anatomical landmarks (Figs. 1 and 2). Foci were localized at the insertion of the two left ventricular papillary muscles and the moderator band on the ventricular free wall; all nodes connecting the structure to the free wall were treated as focus (e.g. assigned equal activation timings). On the septal wall, six regions with a radius of 10 mm were selected containing multiple potential foci and per septal region, one focus was selected. At the left ventricular septal wall, one region was localized at the inferior one-third from base to apex of the septal wall and one region was localized at superior one-third from base to apex of the antero-septal wall and two other regions were localized between those locations. At the right ventricular septal wall, the localized region was close to the RV apex and at the middle of the RV septal wall.

Activation sequences were calculated using a myocardial conduction velocity of 0.85 ms^−1^.^10,13,22,33^ To account for increased subendocardial conduction velocities, myocardial conduction velocity in close vicinity (<15 mm) of foci was set at 1.7 ms^−1^.^22,25,33^ Per region, single focus activation sequences were computed with an initial timing ranging between 0-35 ms for the structure regions (e.g. papillary muscles and moderator band) and between 0-25 ms for the septal regions.^10,15,16,18,34,40^ This procedure resulted in nine groups of single focus activation sequences initiated at one of the His-Purkinje associated regions with distinct initial activation timings and were selected based on the best matching computed and recorded BSPM.

Normal ventricular activation is affected by inter-individual diversity in His-Purkinje anatomy and by structural myocardial disease.^6,15^ Structural myocardial disease may affect the number of active foci at the endocardium and consequently the ventricular activation sequence; in right and left bundle branch block less foci are active compared to normal ventricular activation. This diversity was incorporated in multi-wave *i*ECG by automatically testing all 511(=(2^9^)-1 (all foci inactive)) possible permutations of merged single foci activation sequences . Merged activation sequences with a total activation duration >115% and <85% of measured QRS duration were excluded. The activation sequence yielding highest correlation between recorded and computed BSPM was selected as the initial estimation.

#### Optimization Procedure

The initial estimation is further optimized by matching computed BSPM to recorded BSPM. Therefore, a dedicated Levenberg-Marquardt optimization procedure was used.^27^ The surface Laplacian of the activation times was used as regularization-operator and was performed through the iterative minimization of:

1 $$\mathit{arg}{min}_{\delta }\left({\Vert V-\phi (\delta )\Vert }_{F}^{2}+{{\mu }^{2}\Vert L\delta \Vert }_{F}^{2}\right)$$

with computed BSPM (ϕ), based on LAT (δ), ϕ is minimized to the recorded BSPM (V) by iteratively adjusting δ. The operator L represents the numerical form of the surface Laplacian operator; by minimizing ${\Vert L\delta \Vert }_{F}^{2}$ a spatially smooth solution is promoted. The regularization parameter ${\mu }^{2}$ was set to a very small value $5\cdot {10}^{-6}$ mV^2^ms^2^m^−2^ and chosen such that the optimized activation sequence was regularized to empirically to correspond to realistic smoothness.^35^ We refer to the discussion for the setting of the regularization parameter. A maximum of 25 iterations was needed to optimize δ.

### Quantitative Analysis

Ventricular activation timing maps were displayed from early (red) to late (blue) activation. Recorded BSPM were compared to the computed BSPM by means of the Pearson’s correlation coefficient (CC) and the relative difference. The relative difference is calculated by computing the Frobenius normal form of the difference between estimated and input BSPM divided by the Frobenius normal form of the recorded BSPM.

Per surface, estimated activation maps were compared to invasive activation maps by calculating inter-map CC (Pearson) and mean absolute difference in LAT. Therefore, estimated ventricular activation maps were timing-referenced to the same timing-reference used during the invasive procedure. Per triangle, the myocardial conduction velocity was calculated using the triangulation technique.^4^ In short, the location and activation timing of the three nodes forming one triangle at the surface were used. The average conduction speed and direction can then be determined, assuming that the wavefront is locally planar, and moves with constant speed in the plane of the triangle. A detailed description of the method is explained in the supplementary materials (Page 1). Velocities above 5 mm/ms were excluded from analysis. For both *i*ECG methods, the computation time was determined. Values are displayed using mean ± standard deviation or using median [range] where appropriate.

## Results

### Invasive LAT Mapping Procedure

**Figure 3:**
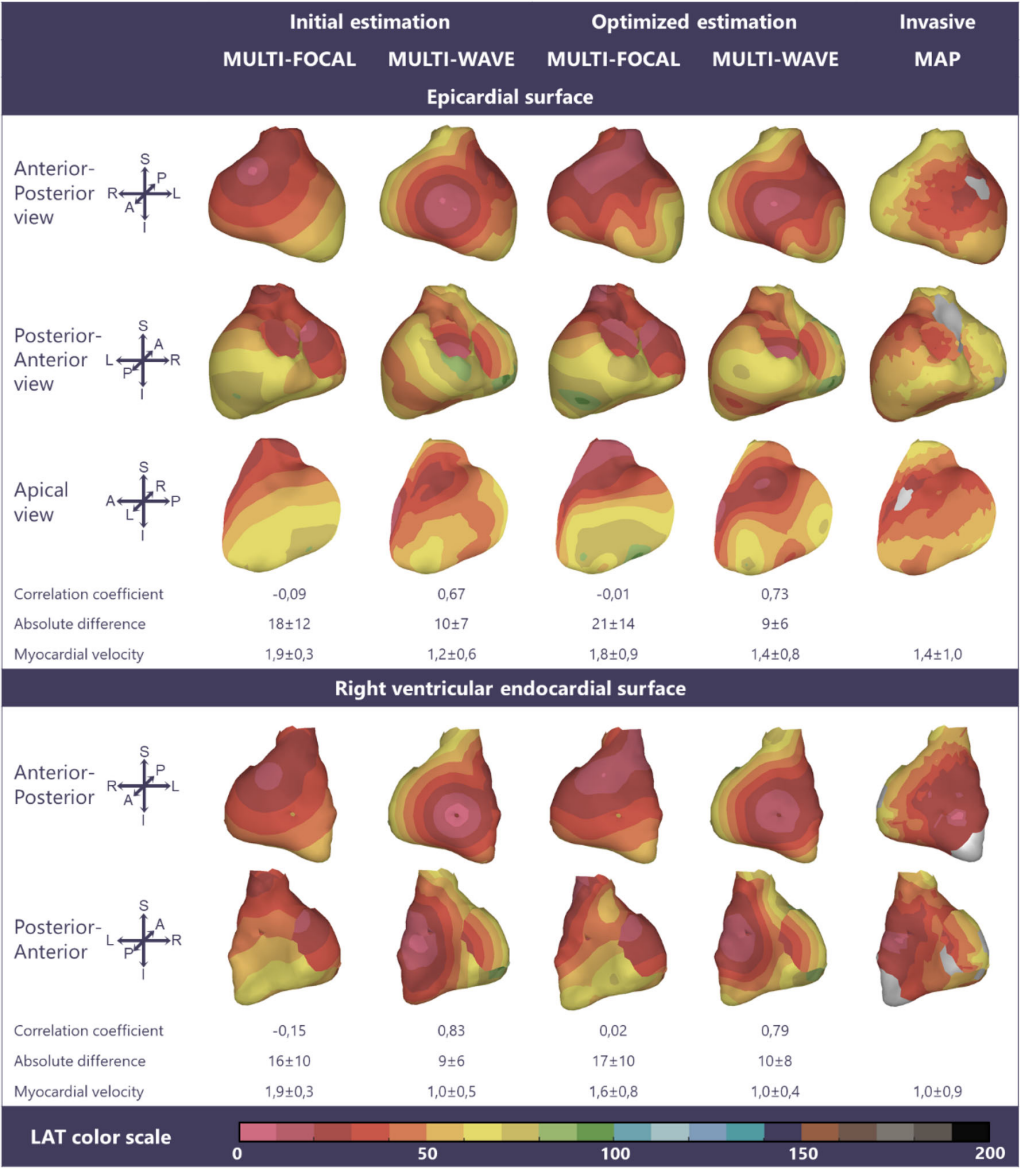
Multi-focal *i*ECG, multi-wave *i*ECG and invasive activation maps of the epicardial and right ventricular endocardial surface (QRS 90 ms) from red (early) to purple (late). The multi-focal *i*ECG map shows one prominent initial site of activation, multi-wave iECG map shows six and is comparable to the invasive map. Inter-map correlation coefficient (CC), absolute difference (ms), LAT time range (ms) and estimated myocardial conduction velocity (mm/ms) are displayed comparing *i*ECG to invasive LAT maps. CC and relative difference between recorded and computed BSPM were 0.99 ± 0.00 respectively 0.15 ± 0.00 for multi-focal *i*ECG and 0.98 ± 0.00 respectively 0.15 ± 0.00 for multi-wave *i*ECG.

**Figure 4:**
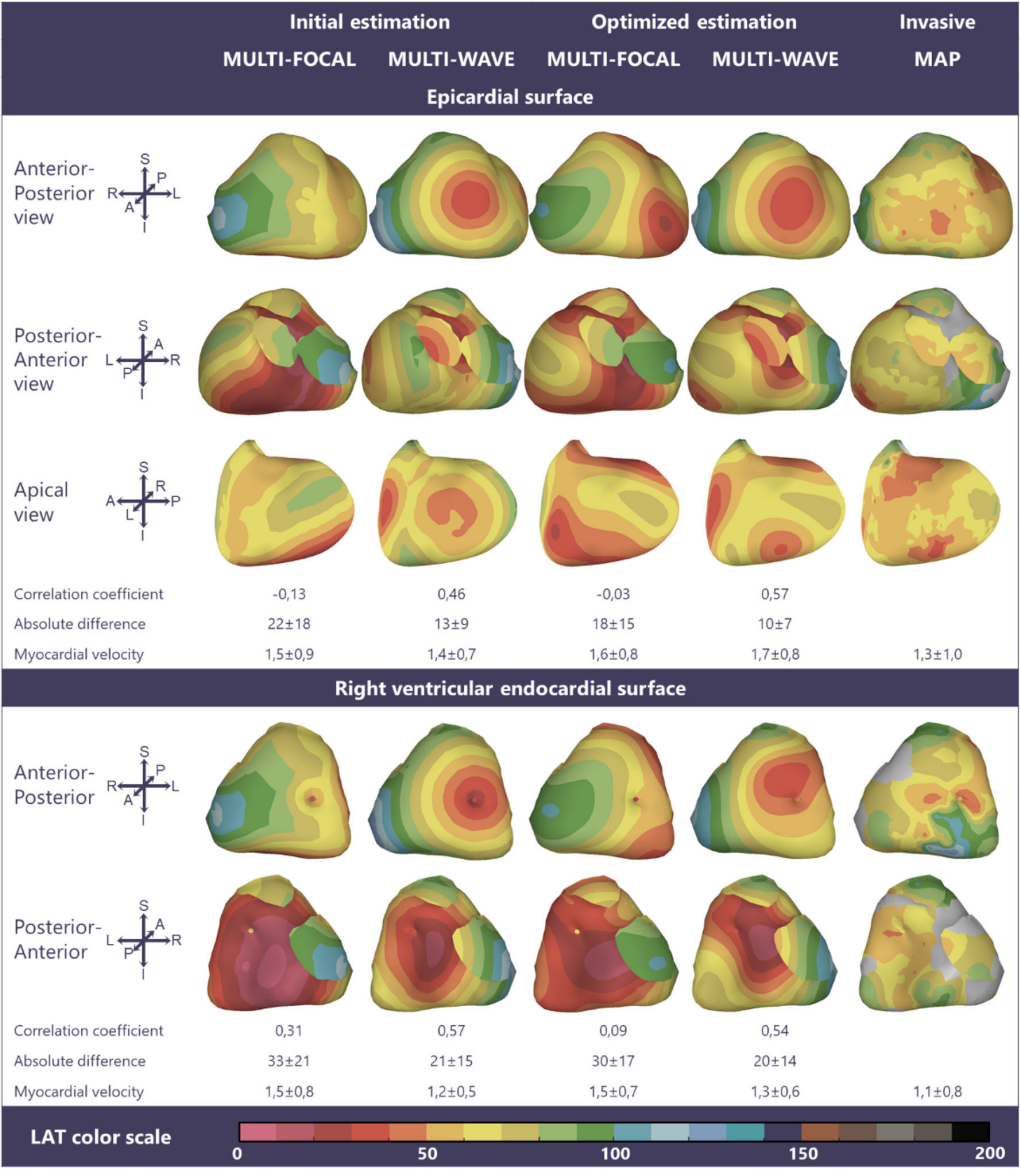
Multi-focal *i*ECG, multi-wave *i*ECG and invasive activation maps of the epicardial and right ventricular endocardial surface (QRS 104 ms) from red (early) to purple (late). Inter-map correlation coefficient (CC), absolute difference (ms), LAT time range (ms) and estimated myocardial conduction velocity (mm/ms) are displayed comparing *i*ECG to invasive LAT maps. CC and relative difference between recorded and computed BSPM were 0.97 ± 0.00 respectively 0.25 ± 0.01 for multi-focal *i*ECG and 0.97 ± 0.00 respectively 0.25 ± 0.01 for multi-wave *i*ECG.

**Figure 5:**
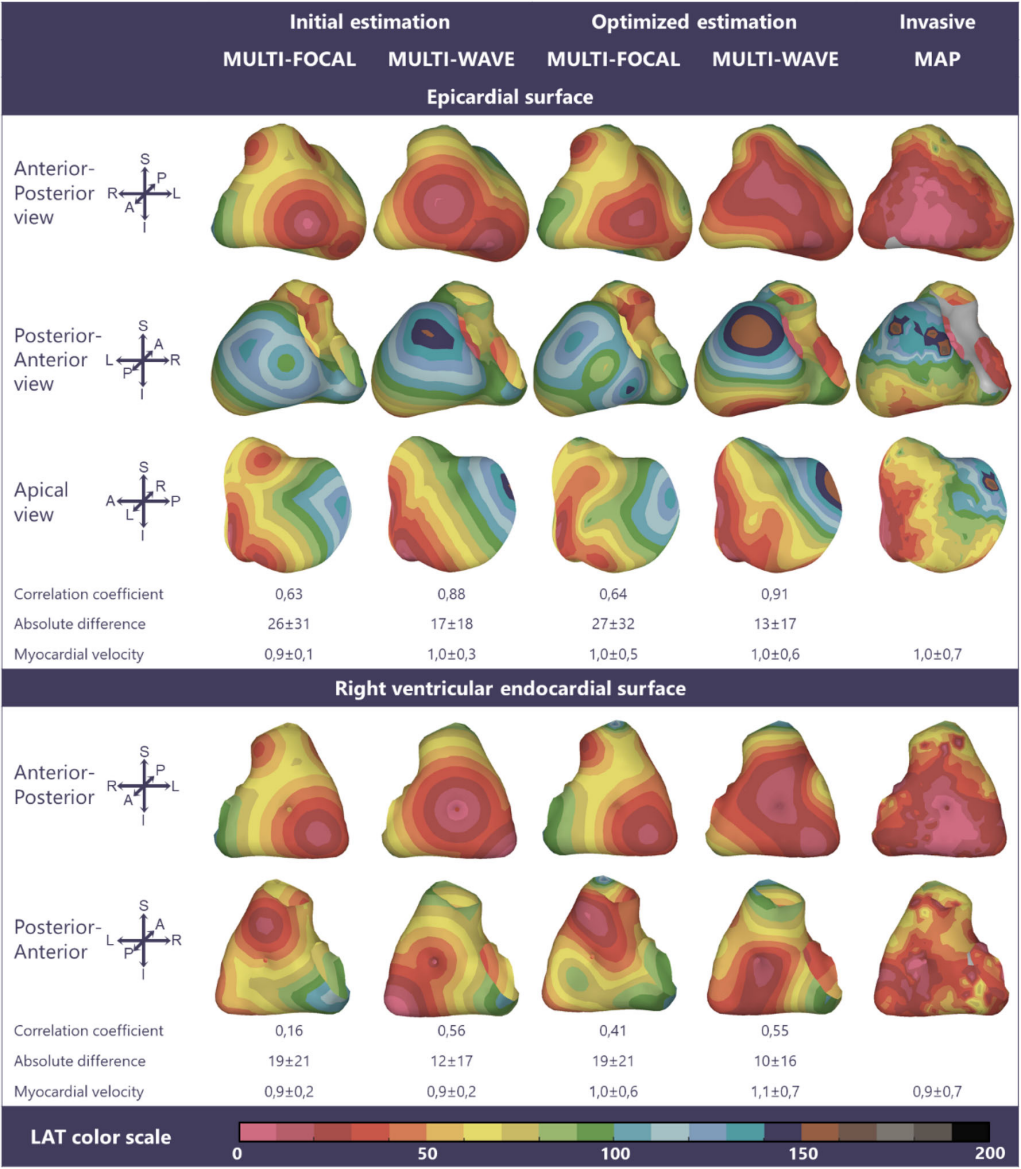
Multi-focal *i*ECG, multi-wave *i*ECG and invasive activation maps of the epicardial and right ventricular endocardial surface (QRS 142 ms) from red (early) to purple (late). Inter-map correlation coefficient (CC), absolute difference (ms), LAT time range (ms) and estimated myocardial conduction velocity (mm/ms) are displayed comparing *i*ECG to invasive LAT maps. CC and relative difference between recorded and computed BSPM were 0.96 ± 0.00 respectively 0.30 ± 0.01 for multi-focal *i*ECG and 0.95 ± 0.00 respectively 0.32 ± 0.01 for multi-wave *i*ECG.

**Figure 6:**
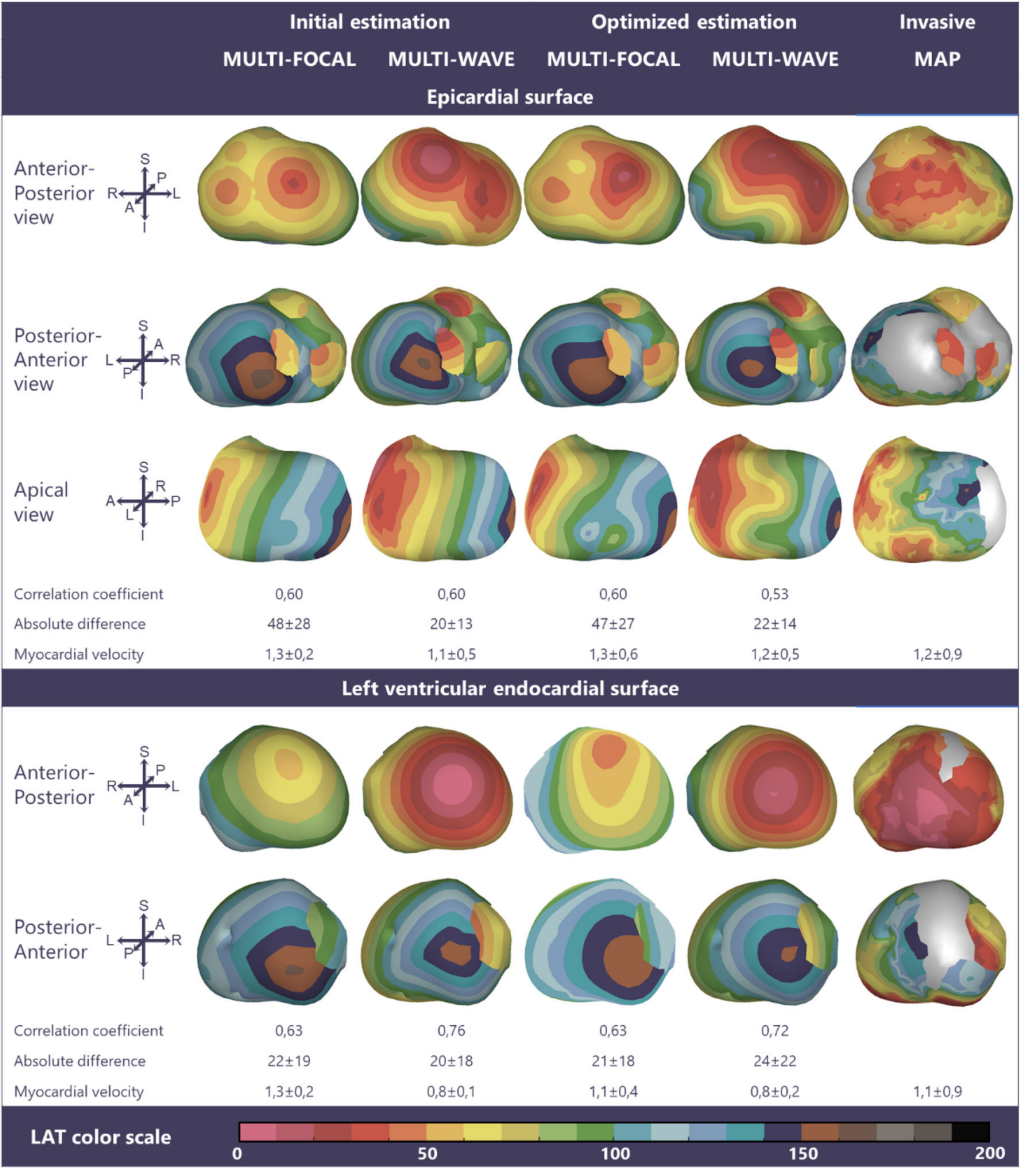
Multi-focal *i*ECG, multi-wave *i*ECG and invasive activation maps of the epicardial and right ventricular endocardial surface (QRS 162 ms) from red (early) to purple (late). Inter-map correlation coefficient (CC), absolute difference (ms), LAT time range (ms) and estimated myocardial conduction velocity (mm/ms) are displayed comparing *i*ECG to invasive LAT maps. CC and relative difference between recorded and computed BSPM were 0.94 ± 0.00 respectively 0.35 ± 0.01 for multi-focal *i*ECG and 0.93 ± 0.00 respectively 0.38 ± 0.01 for multi-wave *i*ECG.

EAM was performed in one subject who had symptomatic premature ventricular contractions originating in the right ventricular outflow tract without structural heart disease (male, 21 years, QRS duration 90 ms, Fig. 3), the three other subjects had recurrent ventricular tachycardias with underlying arrhythmogenic cardiomyopathy (male, 59 years, QRS duration 104 ms, Fig. 4) or dilated cardiomyopathy (female, 65 and 61 years QRS duration 142 and 162 ms, Figs. 5 and 6). In three subjects (Figs. 3, 4, and 5) the epicardium and the right ventricular endocardium were mapped, in the last subject (Fig. 6) the epicardium and the left ventricular endocardium were mapped.

Invasive maps consisted of a mean of 5140 ± 1865 epicardial annotated electrocardiograms and 1476 ± 368 endocardial annotated electrocardiograms. LATs were projected onto the subject specific imaging-based ventricular model. A mean of 73 ± 8% of the epicardial surface and 61 ± 12% of the endocardial surface was mapped with a mapping density (annotations/mm2) of 19.5 ± 7.1 for the epicardial surface and 11.8 ± 3.3 for the endocardial surface. In all subjects, the epicardial surface was mapped. In three subjects, the right ventricular endocardial surface was mapped and in the other subject, the left ventricular endocardial surface was mapped.

### Invasive Versus iECG Local Activation Timing Maps

**Figure 7:**
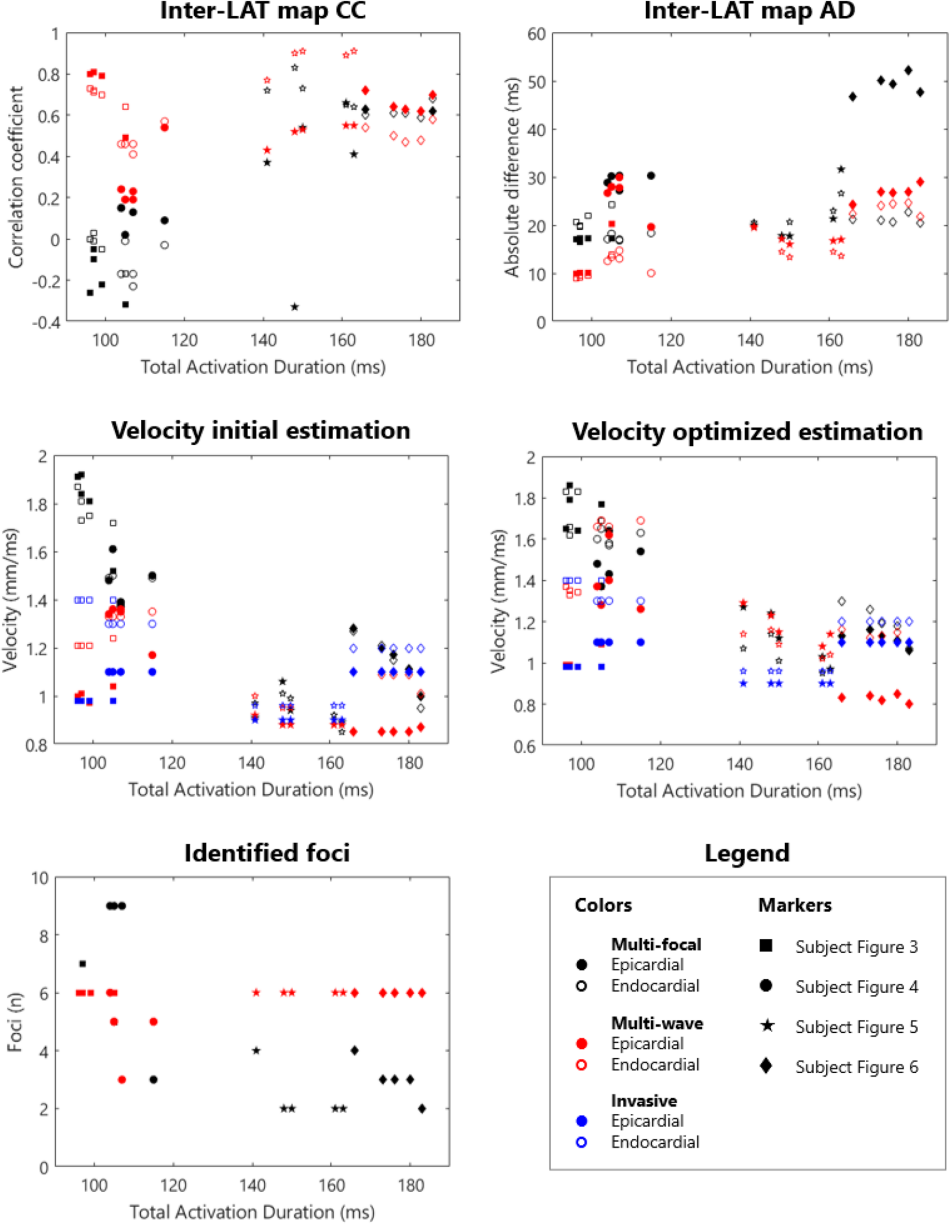
Quantitative comparison of multi-wave, multi-focal and invasive LAT maps. Diamonds represent the epicardial conduction velocity and dots the endocardial conduction velocity; the black color denotes the multi-focal *i*ECG method, the red color the multi-wave *i*ECG method and the blue color the invasive. Upper row: Inter-map correlation coefficient (CC, left) and inter-map absolute difference (right) per subject per plotted beat plotted against QRS duration. With increasing QRS duration, the multi-focal method improves in CC. Middle row: Myocardial conduction velocity estimated from the local activation timing (LAT) maps before (left) and after (right) the optimization procedure per *i*ECG method and for the invasive study per myocardial surface. Bottom row: The number of identified foci in the initial estimation (left) and the legend of all displayed plots (right).

In all four subjects, ventricular activation was estimated with both *i*ECG methods and compared to invasively measured LAT (Figs. 3, 4, 5, and 6). Median epicardial inter-map CC of estimated multi-wave *i*ECG versus multi-focal *i*ECG was 0.61[0.41,0.91] versus 0.31[− 0.23,0.83] and median endocardial inter-map CC was 0.54[0.19,0.81] respectively 0.22[− 0.13,0.64]. With increasing QRS duration, inter-map CC stayed the same for multi-wave *i*ECG, whereas it increased for multi-focal *i*ECG (Fig. 7, upper row, left panel). Inter-map CC, absolute difference, number of breakthroughs per surface and myocardial conduction velocity for all subjects are displayed in Table 1 (inter-map comparison). With shorter QRS durations, myocardial conduction velocity remains constant in multi-wave *i*ECG, whereas it increases in multi-focal *i*ECG (Fig. 7, middle row, left panel). The optimization procedure did not always improve inter-map CC and absolute difference for both multi-wave and multi-focal *i*ECG (Table 1).

**Table 1:** Inter-map comparison.

	Multi-focal	Multi-wave	Invasive
Correlation coefficient			
Epicardial	0.31[− 0.23,0.83]	0.61[0.41,0.91]	
Endocardial	0.22[− 0.13,0.64]	0.54[0.19,0.81]	
Absolute difference (ms)			
Epicardial	21[17,27]	14[9,25]	
Endocardial	27[16,52]	20[10,30]	
Myocardial conduction velocity (mm/ms)			
Epicardial	1.4 ± 0.3	1.3 ± 0.2	1.2 ± 0.2
Endocardial	1.4 ± 0.3	1.1 ± 0.2	1.0 ± 0.1
Number of breakthroughs (n)			
Epicardial	2[2,4]	3[2,5]	3[2,3]
Endocardial	1[1,4]	2[1,3]	2[1,3]
Effect optimization procedure			
Correlation coefficient	− 0.01[− 0.17,0.20]	0.03[− 0.11,0.24]	
Absolute difference	0.7[− 3.7,6.8]	0.7[− 4.8,3.0]	

Quantitative inter-LAT map comparison between the invasive local activation (LAT) and the estimated LAT maps using both iECG methods. Values are displayed per surface as median[range] or mean ± standard deviation where appropriate

Computed and recorded BSPM were similar between the two *i*ECG methods (multi-wave vs multi-focal: CC 0.98 ± 0.01 vs 0.98 ± 0.01 and RD 0.17 ± 0.03 vs 0.17 ± 0.04). Per subject standard 12-lead ECGs of the recorded and computed BSPM are displayed in the Supplementary material (Page 2-5). The number of identified foci decreased with increasing QRS duration in multi-wave *i*ECG, but did not in multi-focal *i*ECG (Fig. 7 middle row). Mean computation time was 33 ± 6 seconds for multi-wave *i*ECG and 1014 ± 726 seconds for multi-focal *i*ECG.

## Discussion

Modeling the effect of the His-Purkinje system by incorporating endocardial electro-anatomical structures in the *i*ECG method improved the accuracy of non-invasive estimation of His-Purkinje mediated ventricular activation, especially in the estimation of normal ventricular activation (Figs. 3, 4, 5, 6, and 7). The overall performance of the multi-wave *i*ECG was superior to multi-focal *i*ECG, as is also shown in our previously published study in a larger patient cohort.^26^

### Comparison of EDL-Based iECG

A method to mimic the effect of the His-Purkinje system on ventricular activation should incorporate all possible variations of His-Purkinje mediated ventricular activation; either healthy or diseased. Multi-focal *i*ECG does not take the effect of the His-Purkinje system on ventricular activation into account. In this method, an additive iterative search over the complete myocardium is performed where the ‘fundamental’ activation sequence dominantly determines the final estimation thereby increasing the chance for an erroneous unphysiological activation sequence.^17,23,35^ This algorithm was found to be the most effective in rather monophasic, simple activation patterns like premature ventricular contractions or ventricular tachycardia. In multi-wave *i*ECG, diverse His-Purkinje anatomy was taken into account by the incorporation of subject specific locations of endocardial electro-anatomical structures associated with early ventricular activation. Flexibility was obtained by testing all permutations of the anatomically identified foci. Therefore, multi-wave *i*ECG is more restrained in foci location, which resulted in more realistic estimations of the ventricular activation sequence in normal ventricular activation compared to multi-focal *i*ECG (Figs. 4 and 5). In broad QRS complexes, the performance of multi-focal *i*ECG improved and became equal to the performance of multi-wave *i*ECG, as reflected in inter-map CC and absolute difference (Fig. 7). Thus, the fundamental difference between both methods lies within the first step of the initial estimation; whereas multi-focal *i*ECG identifies an activation sequence with one focus best explaining the recorded BSPMs, multi-wave *i*ECG provides an estimation with multiple foci (Fig. 2).

### Modeling the His-Purkinje System

In our study, an anatomy-based model of the His-Purkinje system was used for the estimation of both the initial sites of activation and the myocardial conduction velocity. Incorporating a model of the His-Purkinje system has been shown to be essential to reliably simulate sinus rhythm.^5,12,24,39^ In our study, a generic model of the His-Purkinje system was individualized by segmenting anatomical endocardial structures associated with the location of the Purkinje myocardial junctions using patient-specific cardiac imaging. Then, based on the recorded BSPM, location and timing of the initial sites of activation were estimated.

#### Initial Sites of Activation

Physiologically, with shortening of QRS duration, an increased number of His-Purkinje foci will be active, as modeled in multi-wave *i*ECG. With shortening of QRS duration, complexity of the estimation increases due to partial cancellation and amplification of wavefronts represented in the recorded BSPM. Multi-focal *i*ECG is likely to identify an aggregation of near simultaneous wavefronts as the ‘fundamental’ activation sequence. Foci could be localized over the complete myocardium and their initial timings could range within QRS duration, resulting in inadequate estimated activation sequences.^10,15,16,18,34,40^

In His-Purkinje system disorders, the complexity of ventricular activation reduces and QRS duration increases. The performance of multi-focal iECG improved with increasing QRS duration as earlier observed in a EPD based *i*ECG validation study.^9^ However, multi-wave *i*ECG showed that its performance was not affected by QRS duration. The effect of the His-Purkinje system was mimicked through the identification of nine distinct endocardial regions as potential foci. All possible activation patterns were tested by comparing computed to recorded BSPM. Additionally, multi-wave iECG excluded unrealistically long and short total activation durations referenced to QRS duration in the recorded BSPM. This combination resulted in a physiological robust non-invasive estimation of ventricular activation.

#### Myocardial Conduction Velocity

With decreasing QRS duration, the estimated myocardial conduction velocity increased in the initial estimation of multi-focal *i*ECG whereas the number of foci increased in multi-wave *i*ECG (Fig. 7 middle/lower row). The estimation of multi-wave *i*ECG is thus more realistic whilst taking normal physiology into account. In multi-focal *i*ECG, myocardial conduction velocity was estimated between 0.8 and 2.0 mm/ms for the myocardium, increasing with decreasing QRS duration whereas in multi-wave *i*ECG, myocardial conduction velocity was set. The optimization procedure affected the myocardial conduction velocity for both methods to some extent (Fig. 7 middle row).

Values up to 2.0 mm/ms equal the conduction velocity of Purkinje fibers and may be observed in regions with a high density of Purkinje-myocardial junctions^25,33^, but are physiologically unrealistic for the normal myocardium. The estimated myocardial conduction velocities used for the multi-focal initial estimation may be physiologically realistic if ventricular activation is initiated by one focus, as the estimated myocardial conduction velocity was matched to measured QRS duration. If multiple foci contribute to the short QRS duration, myocardial conduction velocity increases in multi-focal *i*ECG, which is physiologically unrealistic. Selecting nine foci as starting point of ventricular activation remains a simplification of the true dense distribution of Purkinje-Myocardial junctions. To account for the effect of this dense distribution, conduction velocity was doubled in the regions directly around foci in multi-wave *i*ECG.

### Myocardial Conduction Velocity Estimation

In this study, the surface myocardial conduction velocity was computed (Fig. 7 middle row). In the areas of breakthroughs at the epicardium, surface conduction velocity may seem to be increased as the wavefront moving from the endocardium to the epicardium almost simultaneously activates the epicardium. However, in endocardial breakthrough regions the dense distribution of Purkinje-Myocardial junctions is active which may realistically contribute to rapid activation of the myocardium. To correct for seemingly increased conduction velocities due to simultaneous breakthroughs at multiple nodes, estimated values above 3 mm/ms were excluded from analysis.

### Comparison to EPD Based iECG

As described by Duchateau *et al*^9^, the performance of EPD based *i*ECG during sinus rhythm is poor (inter-map CC: 0.03 ± 0.43, absolute difference: 20.4 ± 8.6), especially in narrow QRS complexes. With increasing QRS duration, the performance of the method improved. The performance of multi-wave *i*ECG showed a higher overall performance as reflected in inter-map CC and absolute difference and the performance of method seemed to be unaffected by QRS duration.

In contrast to EPD based method, the EDL based method defines the local source strength proportional to the transmembrane potential at both epicardium and endocardium instead of local electrograms at solely the epicardium. For both source models applies that the underlying inverse problem is ill-posed, i.e. completely different ventricular activation sequences can generate similar BSPM waveforms. Subsequently, the computed BSPM from the EDL based method also depend non-linearly on the activation and recovery timings. To obtain a realistic estimate for ventricular activation and recovery, EDL-based *i*ECG requires an initial estimate which can be based on ventricular electrophysiology, in contrast to EPD-based *i*ECG.^17,35^ In multi-wave *i*ECG a several foci are defined for this His-Purkinje mediated activation, thereby correctly reflecting cardiac electrophysiology.

### Optimization Procedure

Due to the non-linear relation between activation time and simulated potentials, the EDL based *i*ECG requires an initial estimation which is then mathematically optimized by minimizing the differences between recorded and computed BSPM by tuning LAT regularized by the surface Laplacian. The optimization procedure both negatively and positively affected the inter-map correlation and absolute differences as compared to the invasive maps. Thus, the initial estimation will not extremely change, meaning that no foci will appear or disappear as an effect of the optimization procedure. However, by optimizing the LAT, modeled local conduction velocity is affected thereby possibly negatively affecting the agreement between the invasive and non-invasive maps. Furthermore, due to proximity effects, wavefronts traveling close to electrodes pose a larger effect in the optimization procedure compared to wavefronts traveling at the posterior side of the heart. The results in this paper thus emphasize both the need for a physiologically realistic initial estimation and necessity of an electrophysiological based regularization of the optimization procedure.^8^

In comparison to other EDL-based iECG methods, we used a lower value for our regularization parameter, meaning that the optimization procedure is less regularized by the surface Laplacian. We tested values between 1.5 × 10^−4^, as used in previous studies^35^, and 5 × 10^−8^ and we observed that the optimized maps did not differ when using a higher vs lower value. As with the decrease of the regularization parameter the optimized activation sequence remained equal, the initial estimation thus needs less physiological regularization.

### Computation Time

A large difference in computation time was observed between the two *i*ECG methods due to the difference in the selection of the initial estimation. This is mostly caused by limiting the search space to identify foci by matching computed to recorded BSPM. Thus, besides the fundamental difference in the methodology to select the initial sites of activation, also the reduction in the computation time favors multi-wave *i*ECG for clinical implementation.

### Limitations

In both multi-focal *i*ECG and multi-wave *i*ECG conduction velocity is assumed to be only affected by anisotropy. The presence of structural heart disease, as present in the included subjects, on the estimation of both *i*ECG methods was not assessed, but may be of great importance as conduction velocity is affected by the presence of abnormal myocardium and fibrous tissue. If local cardiac remodeling is present, a fixed conduction velocity in the *i*ECG method is physiologically not realistic. Therefore, future research will focus on the incorporation of abnormal myocardium in the cardiac source model and the effect of local cardiac remodeling on cardiac conduction velocity and multi-wave *i*ECG estimations. Structural information about the cardiac tissue will then be obtained from dedicated cardiac imaging, and per underlying substrate the appropriate modeling technique will be investigated.

In this study, extensive invasive mapping was used as the gold standard sequentially recorded beats and electrograms are used to estimate LAT. During both the invasive maps and the selection of the beats from the BSPM signals, no respiratory gating was used, possibly resulting in error caused by respiration. Visual comparison of the maps also shows a distinct difference between the invasive and non-invasive maps, where the non-invasive maps are more smoothed compared to the more speckled invasive maps. From the LAT maps Durrer presented, a smoother pattern is expected (like the *i*ECG estimation), but other invasive LAT maps presented in quantitative *i*ECG comparison studies and invasive studies show this more speckled pattern.^9,32,40^ This difference is most likely caused by the density of obtained LAT measurements; the maps of Durrer *et al* are constructed using a large inherent smoothing pattern, due to the sparsity of the number of needles used to obtain the LAT. However, also both inaccuracy in the invasive mapping system or more physiologically based variation in the activation of (diseased) myocardial tissue, may contribute to this speckled pattern. The contribution of these factors is however yet unknown and should be verified as this fundamental difference in pattern will always severely affect presented inter-map correlations and absolute differences.

The His-Purkinje model used in this study can be optimized to the specific patient, however, it remains a crude representation of true cardiac anatomy and electrophysiology. In the model one node surrounded by a region of increased conduction velocity was used to simulate Purkinje-myocardial junction. However, in reality, these junctions are a much more complex system. Therefore, and due to the incompleteness of specifically the endocardial maps, we were not able to compare the EAM mapped focal sites to our iECG estimated focal sites.

## Conclusion

Modeling of the effect of the His-Purkinje system in our novel multi-wave *i*ECG method provides a physiologically robust estimation of the ventricular activation sequence even in normal (narrow QRS) ventricular activation. The computation time required by multi-wave *i*ECG was short, crucial for clinical use. Multi-wave *i*ECG might thus enable the identification and progression of arrhythmogenic substrates in patients with structural heart disease. Future research will be directed towards the combination of the novel His-Purkinje model and the incorporation of myocardial tissue characteristics, e.g. scar, to improve our *i*ECG method.

## Supplementary Information

Below is the link to the electronic supplementary material.

Supplementary file1 (DOCX 442 kb)

## Abbreviations

BSPM: Body surface potential map
CC: Correlation coefficient
CT: Computed tomography
EAM: Electro-anatomical mapping
ECG: Electrocardiography
EDL: Equivalent double layer
EPD: Equivalent potential distribution
FRA: Fastest route algorithm
*i*ECG: Inverse electrocardiography
LAT: Local activation timing
LBBB: Left bundle branch block
RBBB: Right bundle branch block
