# Supplementary material for: Modeling the His-Purkinje Effect in Non-invasive Estimation of Endocardial and Epicardial Ventricular Activation
Source: Ann Biomed Eng. 2022 Jan 24;50(3):343–59. doi: 10.1007/s10439-022-02905-4 (PMC8847268; doi:10.1007/s10439-022-02905-4)
Supplement: Supplementary file 1 — Supplementary file1 (DOCX 442 kb) [file 10439_2022_2905_MOESM1_ESM.docx]

**Local myocardial conduction velocity estimation**

We estimated the myocardial conduction velocity in this study using the triangulation method. The technique allows for the estimation of conduction velocity without requiring constraints based on the spacing and distribution of the nodes of the cardiac mesh. Especially for the sometimes incomplete invasive maps with missing data, this allows for local conduction velocity estimation without requiring also large amounts of surrounding data.

In short, the rules of trigonometry are used to associate the location (coordinates) and LAT to the local conduction velocity. The local conduction velocity is then determined by the three points of a triangle, assuming the wavefront is locally planar.

In the diagram below the computation is displayed schematically together with the used formulas. First θ is estimated, which is the angle at the node of earliest activation (O) of the triangle. Then the angle α is estimated, taking into account the 1) the difference in activation times between nodes, the distances between nodes and the angle between the two edges. The angle α then gives the direction of activation at the current triangle. Then the velocity over the triangle can be estimated by solving the equation with the known edge length, angle α and the time between the two nodes.


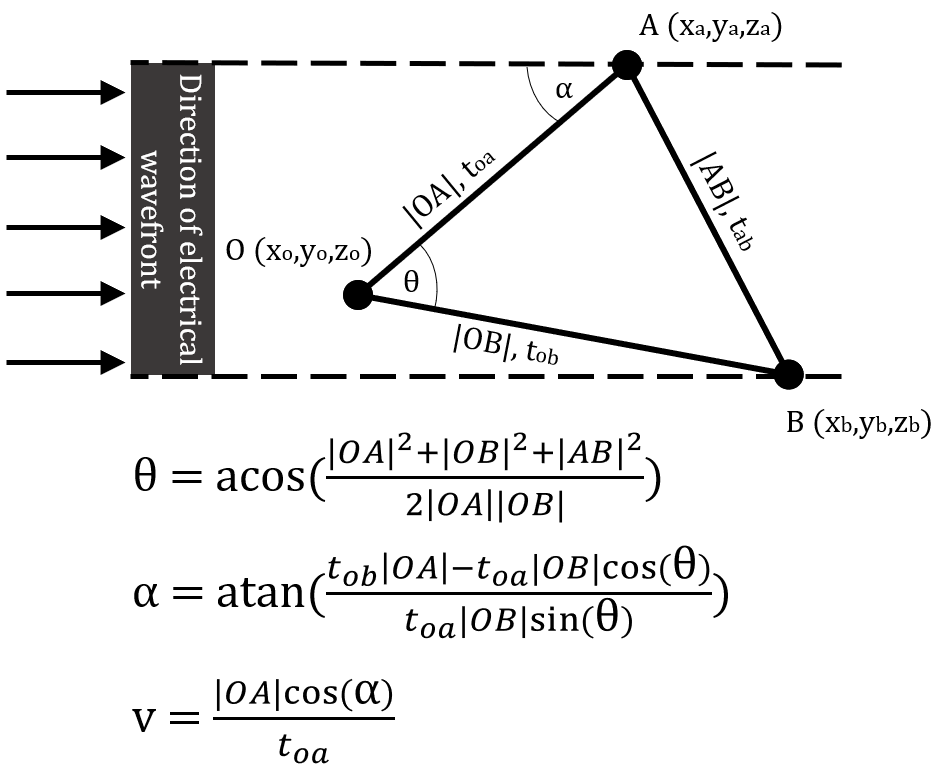


**Supplementary figures**


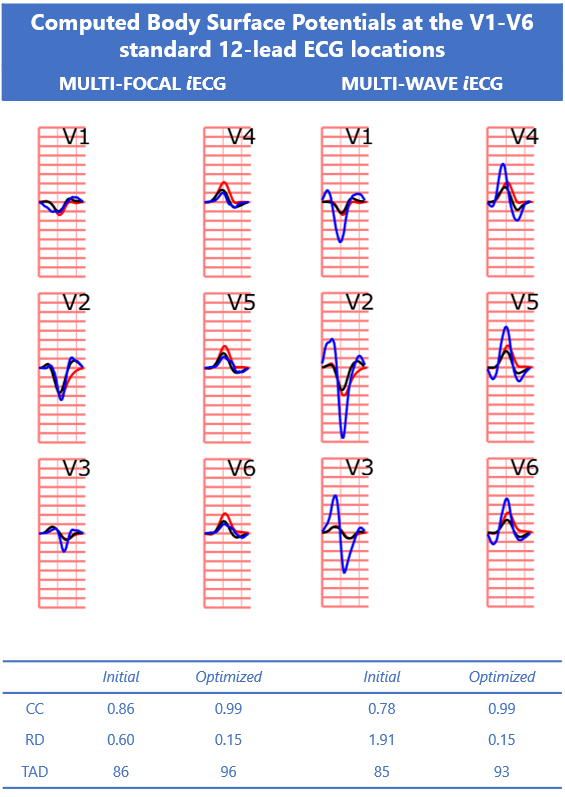


**Supplementary Figure 1: Inter BSPM comparison.** *Precordial leads V1-V6 of the recorded BSPM (red), computed initial BSPM (blue) and computed optimized BSPM (black). The* ***measured QRS duration*** *of the subject is* ***90 ms****. Leads are displayed for the multi-focal iECG method (left) and multi-wave iECG method (right).* *The activation sequence displayed in* ***Figure 3*** *of the manuscript is used to compute the initial and optimized BSPM. In the figure, paper speed is 50 mm/s and amplification is 1 mm/mV. Correlation coefficient (Pearson’s, CC) and relative difference (RD) between computed and recorded BSPM are displayed per method and the estimated total activation duration (TAD) beneath the displayed computed and recorded BSPM.*


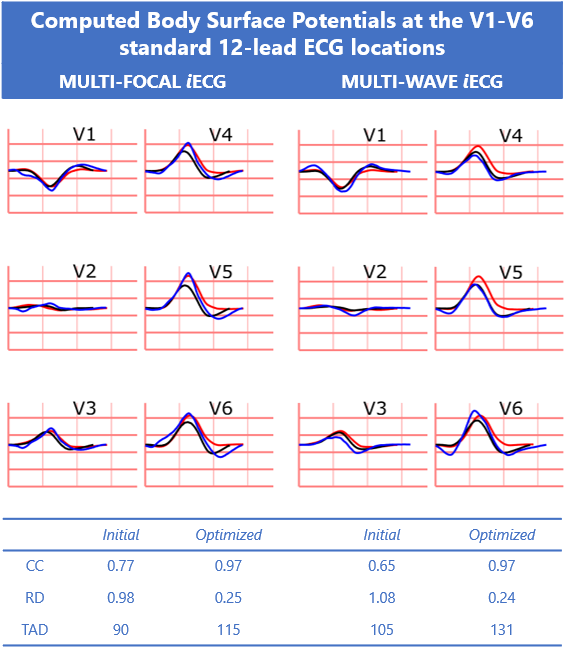


**Supplementary Figure 2: Inter BSPM comparison.** *Precordial leads V1-V6 of the recorded BSPM (red), computed initial BSPM (blue) and computed optimized BSPM (black). The* ***measured QRS duration*** *of the subject is* ***104 ms****. Leads are displayed for the multi-focal iECG method (left) and multi-wave iECG method (right).* *The activation sequence displayed in* ***Figure 4*** *of the manuscript is used to compute the initial and optimized BSPM. In the figure, paper speed is 50 mm/s and amplification is 1 mm/mV. Correlation coefficient (Pearson’s, CC) and relative difference (RD) between computed and recorded BSPM are displayed per method and the estimated total activation duration (TAD) beneath the displayed computed and recorded BSPM.*


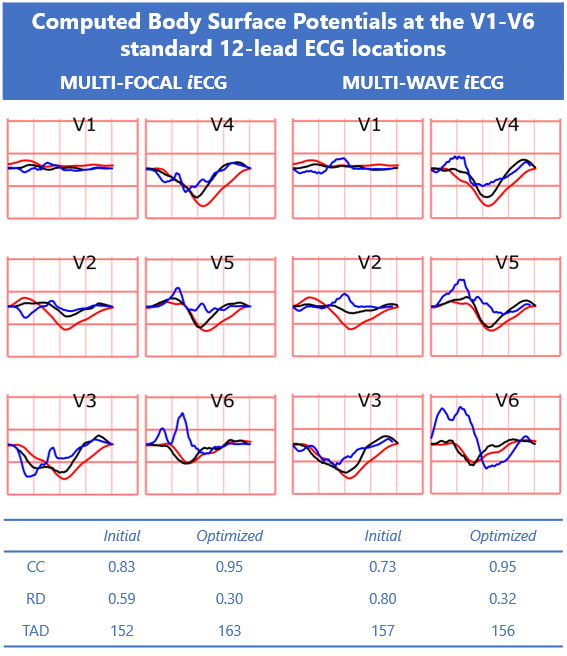


**Supplementary Figure 3: Inter BSPM comparison.** *Precordial leads V1-V6 of the recorded BSPM (red), computed initial BSPM (blue) and computed optimized BSPM (black). The* ***measured QRS duration*** *of the subject is* ***142 ms****. Leads are displayed for the multi-focal iECG method (left) and multi-wave iECG method (right).* *The activation sequence displayed in* ***Figure 5*** *of the manuscript is used to compute the initial and optimized BSPM. In the figure, paper speed is 50 mm/s and amplification is 1 mm/mV. Correlation coefficient (Pearson’s, CC) and relative difference (RD) between computed and recorded BSPM are displayed per method and the estimated total activation duration (TAD) beneath the displayed computed and recorded BSPM.*


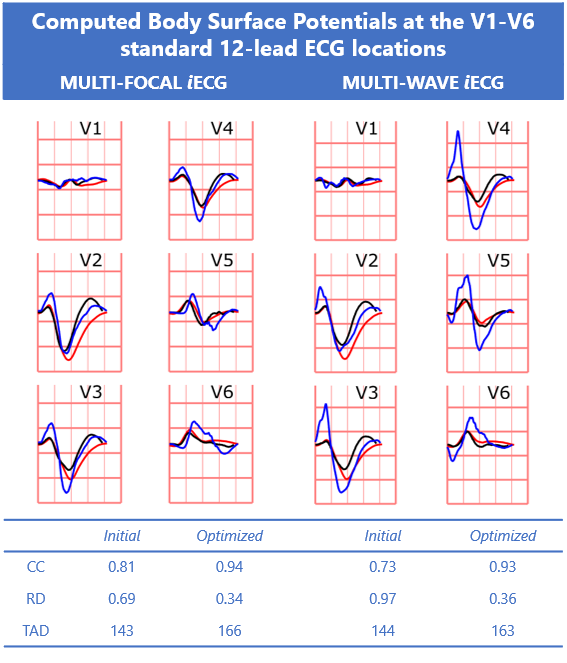


**Supplementary Figure 4: Inter BSPM comparison.** *Precordial leads V1-V6 of the recorded BSPM (red), computed initial BSPM (blue) and computed optimized BSPM (black). The* ***measured QRS duration*** *of the subject is* ***162 ms****. Leads are displayed for the multi-focal iECG method (left) and multi-wave iECG method (right).* *The activation sequence displayed in* ***Figure 6*** *of the manuscript is used to compute the initial and optimized BSPM. In the figure, paper speed is 50 mm/s and amplification is 1 mm/mV. Correlation coefficient (Pearson’s, CC) and relative difference (RD) between computed and recorded BSPM are displayed per method and the estimated total activation duration (TAD) beneath the displayed computed and recorded BSPM.*
